# Value Congruence: A Study of Green Transformational Leadership and Employee Green Behavior

**DOI:** 10.3389/fpsyg.2018.01946

**Published:** 2018-10-09

**Authors:** Xingdong Wang, Kong Zhou, Wenxing Liu

**Affiliations:** ^1^School of Economics and Management, Jiangxi Agriculture University, Nanchang, China; ^2^School of Management, Huazhong University of Science and Technology, Wuhan, China; ^3^School of Business Administration, Zhongnan University of Economics and Law, Wuhan, China

**Keywords:** green transformational leadership, value congruence, green value, employee green behavior, green identity

## Abstract

This study examined the extent to which the impact of green transformational leadership on employee green behavior through follower perceptions of value congruence. Path analyzing on data from 193 subordinate-leader dyads showed that followers’ value congruence with their leader mediated the effects of green transformational leadership on employee green behavior. Results also supported that green identity moderated the indirect effect of green transformational leadership on employee green behavior through value congruence, such that the indirect effect was more positive when green identity was high than when it was low. These findings provided valuable contribution to green transformational leadership, value congruence, and employee green behavior by exploring the relationship between them. Practical implications and directions for future research are also discussed.

## Introduction

Enterprises and individuals are realizing the severity of the environmental problem and calling for establishing a sustainable way of operating. Employee green behavior, a type of pro-environmental action at the workplace (Ones and Dilchert, 2012; Norton et al., 2015), is very important for the organization to realize its goal of environmental sustainability. To promote employee green behavior, it is essential to understand what factors affect such pro-environmental actions and how these effects can be influenced. For example, some scholars have explored the antecedents of employees’ green behavior at the workplace, in terms of corporate strategy (Norton et al., 2017), human resource procedures (Haddock-Millar et al., 2016) and leadership style (Andersson et al., 2013; Chen and Chang, 2013; Graves et al., 2013; Robertson and Barling, 2013; Afsar et al., 2016). In any organizational environment, the characteristics and behaviors of leadership strongly influence the behavior of subordinates (Bass, 1985). Thus, many studies have identified the important effect of green transformational leadership, a leader exhibiting transformation leadership aims to encourage subordinates to engage in pro-environmental behaviors (Robertson and Barling, 2013), on employee green behavior (Chen and Chang, 2013; Robertson and Barling, 2013; Kura, 2016; Mittal and Dhar, 2016). For example, the green transformational leader could promote employees’ green behavior through green organizational identity (Mittal and Dhar, 2016), environmental concern (Kura, 2016) and environmental passion (Robertson and Barling, 2013).

The core of transformational leadership is “values-based leadership” (Shamir et al., 1993; Brown and Treviño, 2009), however, we find little research has explored how green transformational leadership influences employees’ green behavior from a value perspective. The use of green values is at the core of green transformational leadership, therefore the main mechanism of its effect on employee green behavior may be the value channel. Although some scholars have highlighted the importance of green transformational leadership in helping shape employee’s green behavior, we still know little about how organizations can make employees behave greener from the “inside,” i.e., through their values. Given the value-based nature of transformational leadership (Bass, 1985), it might affect the values of subordinates, leading subordinates’ values to become congruent with those of their leaders (Lord and Brown, 2001). It is widely accepted that value is a vital driving factor of individual behaviors, so employees with some green value should be more likely to engage in green behaviors. Hence, it is of great importance to examine the relationship between leadership and employee’s pro-environment behavior from the perspective of personal values.

Values are abstract and they reflect what individuals regard as important in lives. However, knowing what is important does not mean that people would define themselves in that way. For example, maybe you are aware of the importance of protecting the environment, but if you always use the car (perhaps you live far away from the company), you may not define yourself as an environmentalist. We argue that employees’ green identity, the extent which people see themselves as a pro-environmental person (Werff et al., 2014), will influence the degree of their value congruence to their green transformational leader. While interacting with a green transformational leader, a person with green identity can be expected to be sensitive to and more likely to identify with green information regarding the leader’s words and deeds. Thus, we propose that the green identity might moderate the relationship between employees’ perceptions to green transformational leadership and value congruence.

In sum, in accordance with transformation leadership theory and identity theory, this research examines the impact of green transformational leadership on employees’ green behavior via value congruence, and the moderating role of employees’ green identity. Our research extends knowledge about green transformational leadership and employee green behavior by explicitly examining a value-based mechanism that links green transformational leadership to employee green behavior. Although prior research has examined the direct relationship between green transformational leadership and employee green behavior, the underlying mechanisms have remained under-examined, especially from the value perspective. Hence, in this study, we adopt value congruence as a mechanism linking green transformational leadership to employee green behavior. In addition, conceptualizing green identity as a new boundary condition of the effects of green transformation leadership.

## Theory and Hypotheses

### An Overview of Green Transformational Leadership

In the past 20 years, transformational leadership has become a hot topic in the leadership domain (Judge and Piccolo, 2004); many empirical studies have shown that transformational leadership has critical influence on employee behavior (Avolio and Bass, 1988; Bass and Avolio, 1994; Sosik et al., 1998). Transformational leaders inspire employees to shift their attention to the goals conducive to the long-term development of the organization and subordinates, in turn, may internalize the values advocated by their leader and incorporate them into their own self-concept (Shamir et al., 1993; Bono and Judge, 2003). If a transformational leader happens to have green values, we have reason to expect that he/she will exert an influence on his/her subordinates’ green behaviors (Robertson and Barling, 2013). For example, such a leader might motivate subordinates to overcome obstacles by paying more attention to things beneficial to the organizational environment, think about the sustainable development of the organization, and solve environmental problems in an innovative manner; When such a leader makes pro-environmental decisions and conduct pro-environmental behavior in organizations, he/she assumes a pro-environment role model for his/her subordinates. A green transformational leader can raise subordinates’ concerns about environmental issues by establishing good relations with them and then convey them his/her own green values. More than one scholar has highlighted a series of transformational styles exhibited in leaders’ pro-environment behaviors (Chen and Chang, 2013; Robertson and Barling, 2013). Based on Chen and Chang’s (2013) definition of green transformational leadership as well as Bass’s (1998) ideas, “leaders take a series of actions to motivate subordinates to meet the requirements of environmental protection and encourage them to thrive to exceed environmental performance requirements as far as possible.”

### Green Transformational Leadership and Employee Value Congruence

Transformational leaders usually depict a nice and attractive vision to their subordinates and emphasize the congruence between organization’s objectives and their personal values that will imperceptibly influence subordinates to internalize organization’s objectives into their own personal goals and make efforts to accomplish the goal (Shamir et al., 1993; Bono and Judge, 2003). Value congruence refers to the extent to which personal values are in accordance with the surroundings (Burns, 1978; Shamir et al., 1993). It has been used to explain why subordinates are willing to follow their leader and show their loyalty and support (Burns, 1978; Shamir et al., 1993; Klein and House, 1995). Green transformational leaders put more emphasis than usual on the organization’s green vision. They are devoted to the delivery of green values that are beneficial to the organization and environment, making subordinates consider work as a reflection of their own values. In this case, subordinates’internal motivation is involved with the realization of organization’s green objectives rather than external motivation alone. This is because completing these tasks is an embodiment of the realization of their values and these behaviors are in consistence with their self concepts (Shamir et al., 1993). Although green transformational leadership does not change subordinates’ value directly, it effectively links the values of subordinates and the value of work, and continually weakens subordinates’ perceptions of the difference between them, so the individual perception of values tend to become congruent (Klein and House, 1995). Besides, green transformational leaders usually show confidence in themselves and solicitude for subordinates, which drives employees to have a sense of pride and commitment (Dionne et al., 2004), thereby contributing to employees’ perception of value congruence between themselves and their leaders. Following this argument, we hypothesize the following.

**Hypothesis 1:** Green transformational leadership is positively related to followers’ value congruence.

### Mediating Role of Value Congruence

Employees’ values are affected by the environment around them (e.g., the leadership style), which in turn, affects their behavior (Stern et al., 1999). In the field of leadership research, value congruence offers a good explanation for the phenomenon that leader behaviors affect subordinates’ behaviors to a large extent (Hoffman et al., 2011). Therefore, we focus on perceived value congruence as the key mediating process to explain the social influence of green transformational leaders. Values play an important role in the theory of transformational leadership (House, 1996). Within organizations, transformational leaders usually act as effective deliverers of values by assigning tasks and goals, raising subordinates’ self-awareness, and guiding subordinates to be concerned about the long-term goals of organizations (Bass, 1985), which would make their values accepted and internalized by subordinates (Shamir et al., 1993). Specifically, green transformational leaders emphasize organization’s green tasks and environmental values, describe green work ideological terms, and focus on higher-order environmental values. These actions enhance employees’ perception that helping organization to achieve its environmental goals is also a realization of their own values. Thus, subordinates come to see their daily work as congruent with personally held values and thus as more meaningful. Subordinates’ perception of holding the same values as their green transformational leaders should be associated with more employee green behavior.

Once subordinates perceive value congruence with their supervisors, they would be more willing to meet the leader’s requirements and exert themselves further to achieve organizational goals (Lau et al., 2007). Green behaviors are in accordance with organization’s long-term interests. When leaders show green transformational leadership style, subordinates’ attitude toward organizational environment would be more active and they would take the initiative to protect the environment. So green transformational leadership could promote subordinates’ green behaviors by enhancing their perception of value congruence with the leaders. Previous studies have also shown that green transformational leadership could promote employees’ green behavior at work (Graves et al., 2013; Robertson and Barling, 2013). In addition, there is empirical evidence that value congruence affects the effectiveness of transformational leadership. For instance, Jung and Avolio (2000) showed that value congruence mediates the relationship between transformational leadership and its influence on subordinates’ performance. Therefore, we come to the following hypothesis.

**Hypothesis 2:** Value congruence mediates the relationship between green transformational leadership and employee green behavior.

### Moderating Role of Green Identity

Green transformational leaders usually highlight the vital importance of their green values, environment related issues associated with company’s long-term goals as well as their high-level expectations when communicating with subordinates. These expression of environmental values and expectations are essential in subordinates’ value internalization process. Social identity theory points to several kinds of identities (e.g., gender, ethnicity) in individual self-concepts, and identification with a certain role that makes individuals more sensitive to identity-related information (Tajfel, 1979). Therefore, if subordinates have higher identification with environment protection, they will be more sensitive to the environmental information conveyed by the leader and more inclined to internalize leaders’ green value. Green self-identity represents the degree to which the individual regards himself as an environment-friendly person (Werff et al., 2014). Individuals with a high level of green identity probably view themselves and green transformational leaders more as environment-friendly persons. Hence, such employees are more probably to interpret the vision and values conveyed by their green transformational leaders as green, thus experiencing a deeper congruence between their perceived value of the leader and their own. In sum, under the usual assumption of social identity theory, green identity moderates the relationship of transformational leadership and value congruence, which, in turn, moderates the indirect effect of value congruence between transformational leadership and employee green behavior. We therefore hypothesize:

**Hypothesis 3:** Green identity will moderate the mediating relationship of transformational leadership on employee green behavior through value congruence. When employee green identity is high as opposed to low, the positive relationship between green transformational leadership and value congruence, and thus the positive relationship between green transformational leadership and employee green behavior, will be stronger.

## Materials and Methods

### Sample and Procedure

This study was carried out in accordance with the recommendations of the Ethic Committee of fat-food companies in Jiangxi. All subjects gave written informed consent in accordance with the Declaration of Helsinki. The protocol was approved by the Ethic Committee of fat-food companies in Jiangxi. We adopted a two-way questionnaire design by conducting survey in one large manufacturing company in Hubei, China. In the first stage (time 1), we distributed questionnaires to 220 employees, and received 217 effective responses (98.64% response rate) after excluding invalid ones. And then, 1 month later, we distributed questionnaires to 217 supervisor-subordinate dyads (the employees who answered the questionnaires in the first stage and their direct supervisors). In sum, we received 193 effective responses of them (87.73% response rate). Of the 193 respondents, 101 are male (52.3%); the average age is 33.4 years old (*SD* = 7.46) and the average time for employees to work with their leaders is 3.06 years (*SD* = 3.41). As for the education level, average value is 2.15 (*SD* = 0.82), that means, participates have a tertiary education in average.

Specifically, in the first stage (time 1), subordinates were asked to evaluate the transformational green leadership style of their direct supervisors. And 1 month later (time 2), we asked subordinates to evaluate their personal perceptions of value congruence with the leader and green identity while we asked leaders to make appraisals to their subordinates’ green behaviors.

All the measurement scales used in our study were adopted from the west. In order to ensure their effectiveness in Chinese situation, some measures were taken: we first asked several management postgraduate students to conduct a translation- and- back-translation of these scales in a parallel, double-blind way (Brislin, 1980). Subsequently, we discussed the deviation by comparing the translation results with the original sentence and modified our statements properly. After these steps, we invited professors in organizational behavior and human resource management to evaluate the translation of original scales, some culture related usages and ultimately determine the most appropriate Chinese items. Among them, gender is encoded as a dummy variable and “1” represents “male” while “2” represents “female.” In addition, the education level is encode from 1 to 4, rating as “Secondary school (including high school) and below,” “College,” “Undergraduate” and “graduate and above,” respectively. Except for control variables, the measurement of the constructs in this study is by means of “six-point Likert scale from 1 to 6” rating from strongly disagreement to strongly agreement. The definitions and measurements of the constructs in this study are in the following: In order to assess the possibility of non-response bias, a comparison of the early response to those of late response was conducted. Results of variance analysis showed no significant difference between those two groups. Thus, non-response bias was not considered a problem.

### Measures

#### Green Transformational Leadership

Green transformational leadership was measured using the six-item scale developed by Chen and Chang (2013) in Taiwan. Subordinates responded to statements regarding their supervisors’ green transformational behaviors. Sample items include, my leader “encourages the group members to achieve the environmental goals” and “stimulates the group members to think about green ideas.”

#### Value Congruence

We used Brown and Treviño’s (2006) four-item measure of perceived value congruence, namely subordinates’ perceptions of the matching degree with their leaders in values. This scale has demonstrated high factor loadings in Chinese samples (Peng and Lin, 2017), a sample item from this scale is “Since starting this job, my personal value and those of my manager have become more similar.” The subordinates were required to report to what extent they agree that the statements described their perception of their leaders.

#### Employee Green Behavior

We measured employee green behavior using a 7-item scale developed by Robertson and Barling (2013). Sample items from this scale include “This employee prints double sided whenever possible” and “This employee puts compostable items in the compost bin.” In this measure, the leaders were required to report to what extent they agree that the statements described their subordinates.

#### Green Identity

We measured green identity using a 3-item scale (Werff et al., 2014). A sample item from this scale is “I am the type of person who acts pro-environmentally.” The subordinates were asked to indicate the extent to which they agree with the statements.

#### Control Variables

Prior findings have indicated that employee gender, employee age, employee education, and dyad tenure are associated with green behavior (eg., Kim et al., 2017). Therefore, we controlled these four demographic variables.

### Discriminant Validity

We conducted omnibus confirmatory factor analyses (CFAs) to examine the discriminant validity of green transformational leadership, value congruence and green identity. We first ran a measurement model that included three measures (a three-factor model), and then compared it against other models. As shown in **Table 1**, the hypothesized three-factor model fit the data well (χ2 = 124.61, *df* = 62, *p* < 0.001, CFI = 0.95, TLI = 0.93, RMSEA = 0.07) and significantly better compared to other models (Δχ2 differences were significant at *p* < 0.001). The other models had lower CFI and TLI values (ranging from 0.27 to 0.71) and higher RMSEA values (ranging from 0.17 to 0.24). In addition, all factor loadings of the three-factor model were relatively high and significant (*p* < 0.01). Based on these results, we examined the three variables as distinctive constructs.

**Table 1:** Confirmatory factor analyses on the three subordinate-reported variables.

Model	χ^2^	*df*	Δχ^2^*(Δdf)*	CFI	TLI	SRMR	RMSEA
1. Hypothesized three-factor model	124.61	62	-	0.95	0.93	0.06	0.07
2. Two-factor model (GTL and VC are combined)	495.26	64	370.65^∗∗∗^ (2)	0.63	0.55	0.14	0.19
3. Two-factor model (GTL and EI are combined)	408.14	64	283.53^∗∗∗^ (2)	0.71	0.64	0.13	0.17
4. Two-factor model (VC and EI are combined)	413.66	64	289.05^∗∗∗^ (2)	0.70	0.64	0.13	0.17
5. Single-factor model	778.10	65	653.49^∗∗∗^ (3)	0.39	0.27	0.18	0.24


*GTL, Green Transformational leadership; VC, Value congruence; GI, Green identity. All alternative models were compared with the hypothesized three-factor model. CFI, comparative fit index; TLI, Tucker-Lewis index; SRMR, standardized root mean square residual; RMSEA, root mean squared error of approximation. ^∗∗∗^*p* < 0.001.*

## Results

The descriptive statistics and correlations of all study variables are shown in **Table 2**. The results show that green transformational leadership is positively correlated with value congruence (*r* = 0.23, *p* < 0.01) and employee green behavior (*r* = 0.15, *p* < 0.05), and the same to the relationship between value congruence and employee green behavior (*r* = 0.32, *p* < 0.01), which preliminarily supported our predictions.

**Table 2:** Means, standard deviations, and correlations among study variables.

Variable	Mean	*SD*	1	2	3	4	5	6	7	10
1. Gender	1.48	0.50								
2. Age	33.40	7.46	0.13							
3. Education	2.15	0.82	-0.17^*^	-0.48^**^						
4. Dyadic tenure	3.06	3.41	0.01	0.36^**^	-0.24^**^					
5. Green Leader	3.04	0.96	-0.20^**^	-0.06	0.16^*^	0.08	**0.85**			
6. Value congruence	4.07	0.86	0.04	0.03	0.03	0.15^*^	0.23^**^	**0.86**		
7. Green behavior	4.82	0.83	0.12	0.08	-0.04	0.10	0.15^*^	0.32^**^	**0.91**	
8. Green identity	3.71	0.78	0.04	-0.14	0.01	-0.10	0.10	0.05	0.08	**0.87**


*n = 193. Reliability coefficients of our sample are reported along the diagonal in bold. Green leader, green transformational leadership. Education are categorical variables. ^∗^*p* < 0.05; ^∗∗^*p* < 0.01 (two-tailed).*

**FIGURE 1:**
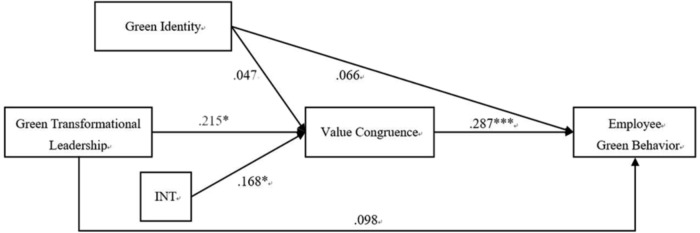
Conceptual model and estimated coefficients. Standardized coefficients are reported. INT, green transformational leadership^∗^Green Identity. ^∗^*p* < 0.05; ^∗∗∗^*p* < 0.001.

**Figure 1** shows the path modeling results for testing our hypotheses (for clarity purposes, the control variables are not shown in this figure). The results show that green transformational leadership significantly predicted value congruence perception (β = 0.215, *p* < 0.01), providing support for Hypothesis 1. The results of bootstrapping analysis (5000 bootstrap samples) confirmed that the indirect effect of green transformational leadership on employee green behavior via value congruence was significant [effect = 0.053, 95% CI = (0.016, 0.113)]. Additionally, the effect of green transformational leadership on employee green behavior was non-significant (β = 0.098, *p* > 0.05). These results indicated that value congruence fully mediated the relationship between green transformational leadership and employee green behavior. Therefore, Hypothesis 2 was supported.

Hypotheses 3 refer to the moderating role of employee green identity. We predicted that employees’ high green identity would strengthen the relationship between green transformational leadership and employee green behavior. Results are reported in **Figure 1** and **Table 3**. **Figure 1** shows that the moderating coefficient for employee green identity is significant (β = 0.168, *p* < 0.05) and **Table 3** shows the conditional indirect effect of green transformational leadership on employee green behavior (through value congruence) with bootstrapping method at two values of employees’ green identity: one standard deviation above the mean and one standard deviation below the mean. Bootstrap CIs show that when employees’ green identity is high, the indirect effect via value congruence is 0.096 [95% CI = (0.034, 0.192)] and is 0.010 [95% CI = (-0.043, 0.069)] when green identity is low. The difference between these two indirect effects was significant, 0.086 [95% CI = (0.012, 0.211)]. Thus, Hypothesis 3 received support.

**Table 3:** Indirect effects of green transformational leadership on green behavior via value congruence at high versus low levels of green identity.

	Path *a* (*SE*)	Path *b* (*SE*)	Indirect effect (95% CI)	Difference in indirect effects (95% CI)
High EI	0.349^∗∗^ (0.104)	0.275^∗∗∗^ (0.071)	0.096^∗^ (0.034, 0.192)	0.086 (0.012, 0.211)
Low EI	0.038 (0.099)	0.275^∗∗∗^ (0.071)	0.010 (-0.043, 0.069)	-


*Unstandardized coefficients and their associated standard errors (SE) are reported in the first two columns, which were used to calculate the indirect effect and their bootstrapped confidence intervals (CIs). ^∗^*p* < 0.05; ^∗∗^*p* < 0.01; and ^∗∗∗^*p* < 0.001 (two-tailed).*

**FIGURE 2:**
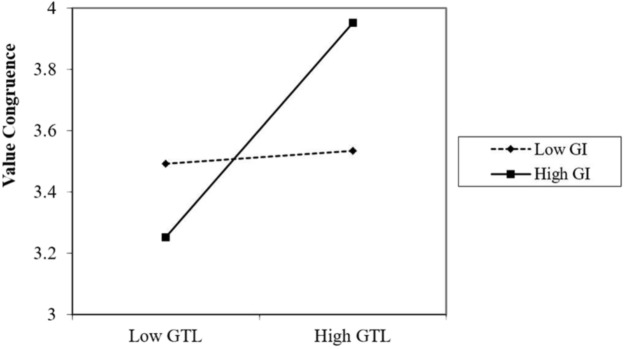
Interaction between green transformational leader and green identity in predicting value congruence. *GTL, Green Transformational leadership; GI, Green identity.*

Using the method recommended by Aiken and West (1991), we plotted the moderating effect using high (1 standard deviation above the mean) versus low (1 standard deviation below the mean) levels of green identity. As shown in **Figure 2**, when green identity is high, the green transformational leadership–value congruence relationship is more positive than when green identity is low.

## Discussion

### Summary of Findings

The purpose of this study was to investigate the relationship of green transformational leadership and employee green behavior from the value congruence perspective. Specifically, we extended previous research by testing a moderated mediation model of green transformational leadership and employee green behavior. Our path analysis results indicated that green transformational leadership is positively related to employees’ perceived value congruence and value congruence mediated by green transformational leadership’s effect on employees’ green behaviors. We have also found an interaction between green identity and green transformational leadership in predicting employee perceived value congruence. In addition, our results have shown that green identity moderates green transformational leadership’s indirect effect on employee green behavior through value congruence. This study has extended previous employee green behavior research by uncovering value congruence as an explanation mechanism that could enhance employee green behavior. These findings have several important theoretical and practical implications for green transformational leadership and employee green behaviors.

### Implications for Theory and Research

There are three theoretical contributions of this study. First, this is one of the first demonstration of the effect of green transformational leadership on subordinates’ green behaviors from a value congruence perspective. The value mechanism is been rooted in the transformational leadership (Shamir et al., 1993; Brown and Treviño, 2009), yet, our understanding of green transformational leaders’ influence on employee green behavior has remained immature. Most of previous studies had adopted the perspective of subordinates’ green consciousness or general internal motivations (e.g., Graves et al., 2013; Robertson and Barling, 2013; Kura, 2016) to explore the internal influencing mechanism of green transformational leadership on employee green behaviors. Our study has demonstrated that green transformational leadership did enhance subordinates’ green behaviors through value congruence, which extends the explanation mechanism of green leadership style on employee’s green behaviors.

Second, this study has indicated that the consistent relation of values between green transformational leader and subordinates didn’t remain constant, especially when there is a striking difference in employee green identities, that is green identity could improve the effect of green transformational leadership on subordinate values. This means that the limitations of green transformational leadership will be exposed when confronting employees who are far from “green.” Individual identity largely affects one’s perception of the outside (Turner et al., 1987). It has been demonstrated in our study that employees with low green identity were less sensitive to green cognition in relation to the surroundings. This finding should be helpful for the scholars to better understand the influencing mechanism of green identity on the employees in an organization.

Furthermore, our study has indicated that in the Chinese culture, green transformational leadership also plays a positive role in promoting green behavior among the subordinates. Previous research samples of green transformational leadership were selected from western developed countries (e.g., Robertson and Barling, 2013; Kura, 2016; Mittal and Dhar, 2016), While few studies have explored such leadership’s effect from developing countries [with the exception of Graves et al. (2013), whose findings are consistent with ours]. For the developing country (e.g., China), the culture and value in the workplace may be largely different from the developed country (e.g., the west countries). The findings of our study validated the effects of green transformational leadership on shaping employee’s green behavior in Chinese culture.

### Practical Implications

The demonstrated influence of green transformational leadership on value congruence highlights the importance of green transformational leadership in creating green value for employee green behavior. We have found that when organizations adopt suitable training programs to cultivate leaders’ green transformational leadership style, the actual benefits they could receive from that afterward were much more than we had expected. In the past, companies usually enhanced green behaviors of their employees through external rewards. But in our study, we have noticed that if the leader could show a greener transformational style, subordinates would more probably accept and internalize these green values of their leader, enhance their own perceptions, and eventually improve spontaneous green behaviors on their part.

### Limitation and Future Research

As with any research, this study also has several limitations. First, the study’s findings have limited generalizability beyond organization studies because the data were obtained from multiple units within the same Chinese company. Future research examining the processes linking green transformational leadership and employee green behavior in different industry sectors or in other cultures should ascertain the extent to which our findings can be generalized. Second, we have suggested that green transformational leadership will assimilate the values of subordinates. However, the cross-sectional data collection methods used in our study are still not enough; more longitudinal designs of the specific formation mechanism of this process are required in the future. For example, the value forming speed may be faster in the early stages of leader-subordinates contact.

Third, our focus in this study has been on employee green behavior. To extend our findings, we encourage future studies to further identify and examine individual and team processes that influence employee green behavior as well as team employee green behavior. Generalized green transformational leadership approaches may include a distinct domain-relevant and employee green behavior-relevant vision that the employees in the team can identify with. Moreover, future multilevel approaches may consider cross-level relationships, thereby highlighting the importance of the situational context for employee green behavior.

Finally, the utility of transformational leadership and its sub-constructs have been critiqued previously (Van Knippenberg and Sitkin, 2013). Our study using a single dimensional measurement and conceptualization on green transformational leadership may limit our theory to determine which green transformational leader behaviors contribute to employee value congruence. However, our scale has been well-validated in previous (e.g., Robertson and Barling, 2013; Kura, 2016) and our study. Future research investigating different discrete green behaviors, such as green vision communication, may enhance our understanding of how green transformational leadership engender employee green behaviors.

## Conclusion

The present study has provided preliminary evidence on the role of green transformational leadership in facilitating employee value congruence, which, at the same time, enhances employee green behavior. The empirical results have supported our hypothesized model, suggesting that green transformational leadership has an indirect influence on employee green behavior through value congruence, and that this mediation effect could be moderated by green identity. We hope that the theory and findings presented in this study have shed new light and will stimulate further theoretical and empirical examination of the link between leadership and employee green behavior.

## Author Contributions

XW, KZ, and WL substantially made contributions to the conception of this research. XW drafted the paper and made contributions to the interpretation of data for this work. KZ made contributions to the analysis and interpretation of data for this work and revised the work critically for important intellectual content. WL made contributions to the acquisition of the data for this work and revised the work critically for important intellectual content. All authors approved the version to be published and agreed to be accountable for all aspects of the work in ensuring that questions related to the accuracy or integrity of any part of the work are appropriately investigated and resolved.

## Conflict of Interest Statement

The authors declare that the research was conducted in the absence of any commercial or financial relationships that could be construed as a potential conflict of interest.
